# Impacts of farming activities on carbon deposition based on fine soil subtype classification

**DOI:** 10.3389/fpls.2024.1381549

**Published:** 2024-05-31

**Authors:** Qiuju Wang, Dongdong Zhang, Feng Jiao, Haibin Zhang, Zhenhua Guo

**Affiliations:** ^1^ Heilongjiang Provincial Key Laboratory of Soil Environment and Plant Nutrition, Heilongjiang Institute of Black Soil Protection and Utilization, Heilongjiang Academy of Agricultural Sciences, Harbin, China; ^2^ Heilongjiang Bayi Agricultural University, Daqing, China; ^3^ Heilongjiang Academy of Agricultural Sciences, Animal Husbandry Research Institute, Harbin, China

**Keywords:** carbon sink, Sanjiang Plain, soil organic carbon, soil bacterial community, land-use model

## Abstract

**Introduction:**

Soil has the highest carbon sink storage in terrestrial ecosystems but human farming activities affect soil carbon deposition. In this study, land cultivated for 70 years was selected. The premise of the experiment was that the soil could be finely categorized by subtype classification. We consider that farming activities affect the soil bacterial community and soil organic carbon (SOC) deposition differently in the three subtypes of albic black soils.

**Methods:**

Ninety soil samples were collected and the soil bacterial community structure was analysed by high-throughput sequencing. Relative changes in SOC were explored and SOC content was analysed in association with bacterial concentrations.

**Results:**

The results showed that the effects of farming activities on SOC deposition and soil bacterial communities differed among the soil subtypes. Carbohydrate organic carbon (COC) concentrations were significantly higher in the gleying subtype than in the typical and meadow subtypes. *RB41*, *Candidatus-Omnitrophus* and *Ahniella* were positively correlated with total organic carbon (TOC) in gleying shallow albic black soil. Corn soybean rotation have a positive effect on the deposition of soil carbon sinks in terrestrial ecosystems.

**Discussion:**

The results of the present study provide a reference for rational land use to maintain sustainable development and also for the carbon cycle of the earth.

## Highlights

- Soil bacteria *RB41*, *Candidatus-Omnitrophus* and *Ahniella* being positively correlated with SOC deposition.
- The effect of farming activities on soil bacterial communities and SOC deposition is different for the three soil subtypes.
- Corn soybean rotation have a positive effect on the deposition of soil carbon sinks in terrestrial ecosystems.

## 1 Introduction

Soil has the highest carbon sink storage in terrestrial ecosystems (Tao et al., 2023; Ma et al., 2024). Human farming activities affect carbon deposition, and soil carbon sinks change with human activities (Wang et al., 2022). Black soils are the most fertile soils, providing most of the world’s food, and there are three famous black soil belts in the world, the Sanjiang Plain in China belong one of them (Wu et al., 2022). The Sanjiang Plain has been under cultivation for 73 years since 1950, which is a very short period of time compared to the process of black soil formation.

**Figure 1:**
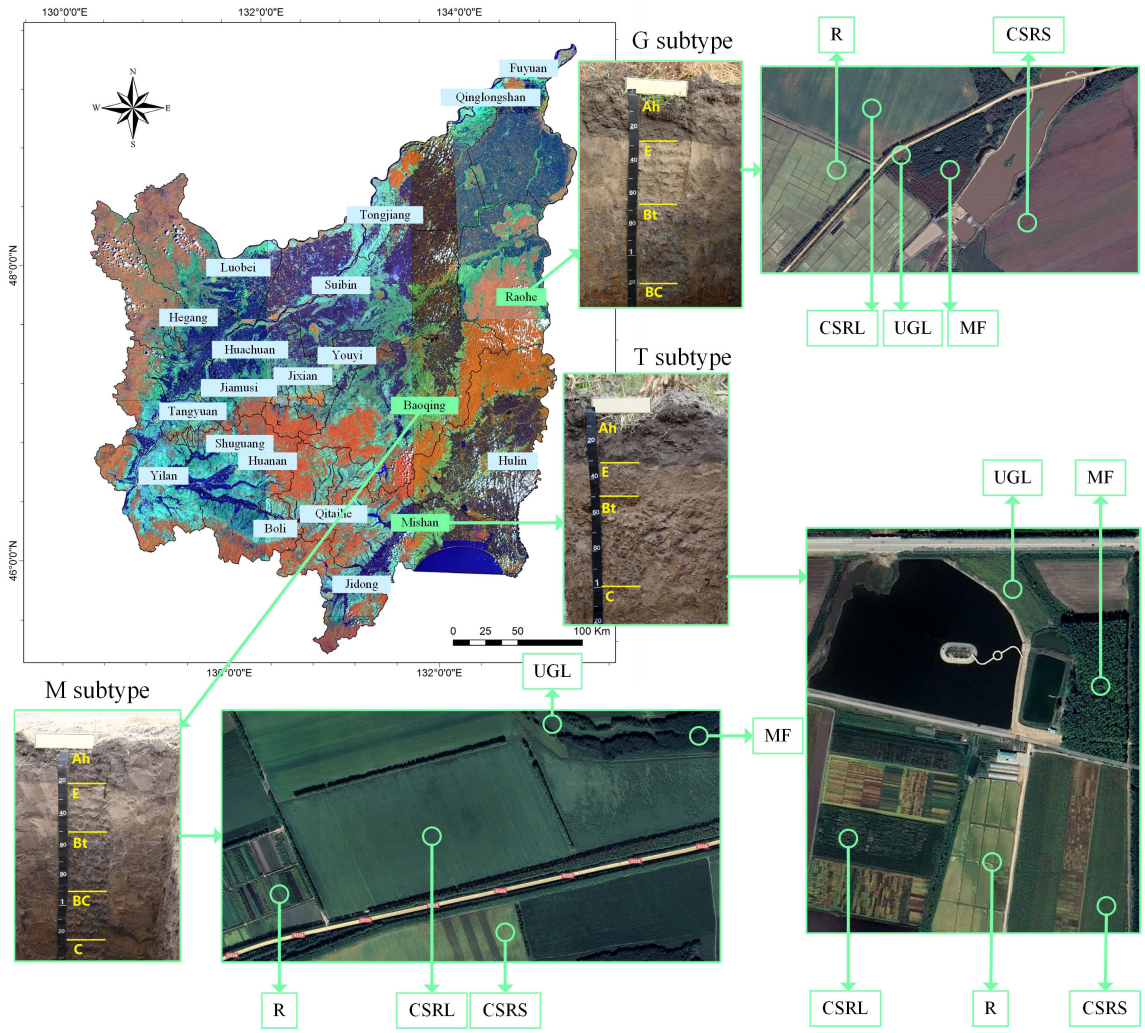
Location of soil samples: Sanjiang Plain is located in the black soil area; Ah represents the plough layer; E represents the albic layer; Bt represents the cohesive sedimentary layer; BC represents the transition layer; and C represents the parent material layer.

All of the cropland in the Sanjiang Plain is located in the black soil zone, where 25% of the soil is albic black soil (also called meadow soil, white pulp soil, albic soil) (Wang et al., 2022). A prerequisite for soil science research is a rigorous soil classification. In this study, we adopted the description of albic black soil (Kumar Sootahar et al., 2019). **Figure 1** shows that albic black soil is a typical classification. The albic black soils are also classified into three subtypes – typical, meadow and gleying – based on their topography, vegetation and soil profile characteristics. The typical subtype is found on undulating slopes and is forested vegetation and deciduous secondary forests, with no induced patches in the profile. The meadow subtype is found on elevated flats and is scrubby meadow miscellaneous grasses, with rust patches seen in the sedimentary layer. The gleying subtype is found in low-lying areas and is marshy grassy vegetation with rust spots in the albic layer. We consider that the soil bacterial communities and soil organic carbon (SOC) deposition of the three subtypes are different.

Soil organic carbon (SOC) deposition in the Sanjiang Plain has been described in a previous study (Wang et al., 2022) but how SOC is formed and deposited into the soil has not yet been determined conclusively (Tao et al., 2023). SOC deposition is important for soil fertility and the ecological environment. Soil bacteria, one of the key biological factors in the SOC cycle, have a significant influence on the transformation and deposition of SOC. Soil microorganisms are mainly involved in the following processes during SOC deposition: (1) bacterial decomposition, where soil bacteria decompose organic matter by secreting enzymes to convert it into small-molecule organic matter (Bardgett and van der Putten, 2014); (2) mineralization, in which inorganic substances in decomposition products are released by soil bacteria to form ions in the soil solution (Jin et al., 2023); and (3) assimilation, where soil bacteria convert organic matter into cellular components for growth and reproduction (Stone et al., 2021). Currently, the commonly used method is high-throughput sequencing technology, which is applied to study soil bacterial diversity, community structure and functional genes to provide a basis for revealing the influence of soil bacteria on organic carbon deposition (Ansari et al., 2024). In addition, stable isotope tracer techniques are used to study the role of soil bacteria in the organic carbon cycle (Stone et al., 2021). Both bacteria and fungi play important roles in soil carbon cycling. However, it has been reported that soil bacteria from three genera absorb approximately 50% of the carbon (Stone et al., 2021), so our experiment only measured soil bacteria.

Soil microorganisms influence carbon accumulation through multiple pathways (Tao et al., 2023). Reports correlating microbes with total organic carbon (TOC), humus organic carbon (HOC), carbohydrate organic carbon (COC) and mineralizable organic carbon (MOC) are limited. Considering that forest soil is also an important part of the soil carbon pool (Chen et al., 2023), we collected manmade forest soil (Poplars, with 4–6 meters row spacings and 2–3 meters individual spacings) and soil from farming activities for comparative analysis. The purpose of this study is to reveal the effect of land use on SOC deposition. And how, exactly, do soil bacteria figure into that? Specifically, this study conducted three comparisons, including two different depths, three soil subtypes, and five ground flora types. The results of the study can provide suggestions for the sustainable development of agriculture, as well as a reference for policies to rationalize carbon emissions.

## 2 Materials and methods

### 2.1 Test soil and sample collection

Soil samples were collected in the period 17–25 April 2023 based on the distribution of the three albic black soil subtypes. **Figure 1** shows that soil samples were collected from Shuangfeng Farm in Mishan City (typical subtype, longitude 131.87, latitude 45.64), Shengli Farm (gleying subtype, longitude 133.77, latitude 47.31) in Raohe County and 852 Farm (meadow subtype, longitude 132.63, latitude 46.23) in Baoqing County. Soil profiles were excavated in multiple locations based on the topographic conditions of albic black soil formation. Three sampling locations were ultimately determined. Excavated profiles identified typical, meadow and gleying subtypes: the typical subtype has a thin black soil layer, mostly deciduous mixed wood secondary forest, with no rust spots in the profile; with the meadow subtype, rust spots are visible in the sedimentary layer; and the gleying subtype has a thicker black soil layer, with rust spots visible in the albic layer.

At every sampling site, soils of five land-use models were collected: uncultivated grassland/meadow (UGL), manmade forest (MF), 8–10 years of corn soybean rotation (CSRS), 15–20 years of corn soybean rotation (CSRL) and rice cultivation of more than 20 years (R). A random five-point sampling method was used, mixed as one sample, and three random replicates of each soil type were collected. Samples were collected at 0–20 cm (shallow, S, plough layer) and 20–40 cm (deep, D, plowpan), respectively; details of the 90 samples and 30 treatments, along with sample names and abbreviations, are given in **Table 1**. Fertilization practices may vary slightly depending on the fertility of the individual plots. We conducted three comparisons, including two different depths (S and D), three soil subtypes (typical, gleiing, and meadow), and five ground flora types (UGL, MF, CSRS, CSRL, and R).

**Table 1:** Details of soil samples.

Treatment no.	Sample no.	Full name	Abbreviation	Total nitrogen (g/kg)	Total phosphorus (g/kg)	Total potassium (g/kg)	Fertilization, tillage, irrigation management (kg/ha)	Average annual production (kg/ha)
1	1-3	Typical corn soybean rotation short-time shallow	TCSRSS	1.83	0.6	20.12	Corn N 16, P 11, K 3, soybean N 3, P 8, K 5	Corn 9500, soybean 2600
2	4-6	Typical corn soybean rotation long-time shallow	TCSRLS	1.63	0.79	18.55	Corn N 15, P 11, K 4, soybean N 2, P 8, K 4	Corn 9350, soybean 2300
3	7-9	Typical rice shallow	TRS	1.59	0.77	18.54	N 8, P 5, K 6	8300
4	10-12	Typical manmade forest shallow	TMFS	1.29	0.66	17.27		
5	13-15	Typical uncultivated grassland/meadow shallow	TUGLS	2.86	0.68	16.59		
6	16-18	Meadow corn soybean rotation short-time shallow	MCSRSS	1.93	0.57	17.63	Corn N 17, P 10, K 5, soybean N 2, P 6, K 3	Corn 9900, soybean 2750
7	19-21	Meadow corn soybean rotation long-time shallow	MCSRLS	1.7	0.84	16.23	Corn N 15, P 9, K 4, soybean N 3, P 8, K 6	Corn 9750, soybean 2500
8	22-24	Meadow rice shallow	MRS	1.58	0.75	16.02	N 7.5, P 6, K 7	8500
9	25-27	Meadow manmade forest shallow	MMFS	1.38	0.7	15.31		
10	28-30	Meadow uncultivated grassland/meadow shallow	MUGLS	1.75	0.62	15.31		
11	31-33	Gleying corn soybean rotation short-time shallow	GCSRSS	1.57	0.7	14.92	Corn N 16, P 9, K 6, soybean N 2, P 7, K 5	Corn 8700, soybean 2000
12	34-36	Gleying corn soybean rotation long-time shallow	GCSRLS	1.55	0.74	15.36	Corn N 15, P 10, K 4, soybean N 3, P 8, K 6	Corn 8500, soybean 1900
13	37-39	Gleying rice shallow	GRS	0.99	0.41	16.56	N 7.5, P 4.5, K 6	7500
14	40-42	Gleying manmade forest shallow	GMFS	1.87	0.45	16.84		
15	43-45	Gleying uncultivated grassland/meadow shallow	GUGLS	2.01	0.75	16.57		
16	46-48	Typical corn soybean rotation short-time deep	TCSRSD	1.51	0.75	15.04	Corn N 16, P 11, K 3, soybean N 3, P 8, K 5	Corn 9500, soybean 2600
17	49-51	Typical corn soybean rotation long-time deep	TCSRLD	1.42	0.81	14.39	Corn N 15, P 11, K 4, soybean N 2, P 8, K 4	Corn 9350, soybean 2300
18	52-54	Typical rice deep	TRD	0.76	0.39	16.65	N 8, P 5, K 6	8300
19	55-57	Typical manmade forest deep	TMFD	1.22	0.33	17.62		
20	58-60	Typical uncultivated grassland/meadow deep	TUGLD	1.95	0.78	14.21		
21	61-63	Meadow corn soybean rotation short-time deep	MCSRSD	1.62	0.69	14.16	Corn N 17, P 10, K 5, soybean N 2, P 6, K 3	Corn 9900, soybean 2750
22	64-66	Meadow corn soybean rotation long-time deep	MCSRLD	1.8	0.79	15.29	Corn N 15, P 9, K 4, soybean N 3, P 8, K 6	Corn 9750, soybean 2500
23	67-69	Meadow rice deep	MRD	2.31	0.9	14.62	N 7.5, P 6, K 7	8500
24	70-72	Meadow manmade forest deep	MMFD	2.86	0.66	13.69		
25	73-75	Meadow uncultivated grassland/meadow deep	MUGLD	1.77	0.62	14.65		
26	76-78	Gleying corn soybean rotation short-time deep	GCSRSD	1.64	0.78	13.48	Corn N 16, P 9, K 6, soybean N 2, P 7, K 5	Corn 8700, soybean 2000
27	79-81	Gleying corn soybean rotation long-time deep	GCSRLD	1.86	0.75	14.83	Corn N 15, P 10, K 4, soybean N 3, P 8, K 6	Corn 8500, soybean 1900
28	82-84	Gleying rice deep	GRD	2.24	0.85	14.36	N 7.5, P 4.5, K 6	7500
29	85-87	Gleying manmade forest deep	GMFD	2.37	0.58	13.91		
30	88-90	Gleying uncultivated grassland/meadow deep	GUGLD	1.31	0.71	16.17		

Treatments: 1–5, typical shallow (TS); 6–10, meadow shallow (MS); 11–15, gleying shallow (GS); 16–20, typical deep (TD); 21–25, meadow deep (MD); 26–30, gleying deep (GD).

### 2.2 Bacterial diversity analysis

Soil samples were cryopreserved in liquid nitrogen and sent to Allwegene Technology (Beijing, China). The genomic DNA of the samples was analysed using an E.Z.N.A. Soil DNA Kit (Omega Bio-Tek, Inc., USA). 16s rDNA libraries were constructed after DNA passed the quality test. Samples were analysed using Illumina NovaSeq6000 (Illumina, Inc.). Linear discriminant analysis of effect size (LEfSe) was performed using Python (v2.7) software for LDA, with the score threshold set at 3. Samples were analysed with a focus on *Bradyrhizobium*, *Acidobacteria* genus *RB41* and *Streptomyces*, three genera associated with carbon deposition (Stone et al., 2021).

### 2.3 Soil organic carbon analysis

The 90 soil samples described above were sent to the laboratory to measure TOC, HOC, COC and MOC according to a previously listed methods (Wang et al., 2022). Briefly, these four organic carbon measurement methods were used in order: oil-bath potassium dichromate oxidation–volumetric; sodium phosphate extraction–potassium dichromate volumetric; constant temperature culture–hydrochloric acid titration; and anthrone colourimetric. Changes in TOC, HOC, COC and MOC were analysed comparatively according to 30 treatments.

#### 2.3.1 Relative changes in SOC

For the 30 treatments (90 soil samples), mean values of the four SOCs (TOC, HOC, COC, and MOC) were calculated. Concentrations of MF, CSRS, CSRL and R were plotted on a heat map in comparison to the SOC concentrations at UGL. For example, the TOC variation of TCSRSS was as follows:


$${\text{TCSRSS}}_{\text{Change TOC}}=\text{Log}(\frac{{\text{TCSRSS}}_{\text{TOC}}}{{\text{TUGLS}}_{\text{TOC}}})$$

#### 2.3.2 Significance analysis of SOC differences

The 30 treatments (90 soil samples) were grouped according to two soil depths, three albic black soil subtypes and five land uses. One-way ANOVA comparisons were performed to analyse the changes in concentrations of TOC, HOC, COC and MOC at the 95% confidence level (STATA, version 15.1). The SOC biodegradability was also calculated and analysed together (Liu et al., 2023b). For example, the SOC biodegradability of TCSRSS was as follows:


$${\text{TCSRSS}}_{\text{SOC biodegradability}}=100\times (\frac{{\text{TCSRSS}}_{\text{MOC}}}{{\text{TCSRSS}}_{\text{TOC}}})\%$$

### 2.4 Correlation analysis between SOC and bacterial concentration

Concentrations of TOC, HOC, COC and MOC were analysed by regression with bacterial operational taxonomic unit (OTU) for the 90 soil samples mentioned above. R software (version 4.1.2) with the “ggplot2” and “dplyr” packages was used. Linear regression was used in this study and an *r* ^2^ value of > 0.5 was considered to be a positive correlation. GraphPad Prism (version 9.0.2) was used to plot the results.

## 3 Results

### 3.1 Farming activities affect the structure of soil bacterial communities

**Figure 2:**
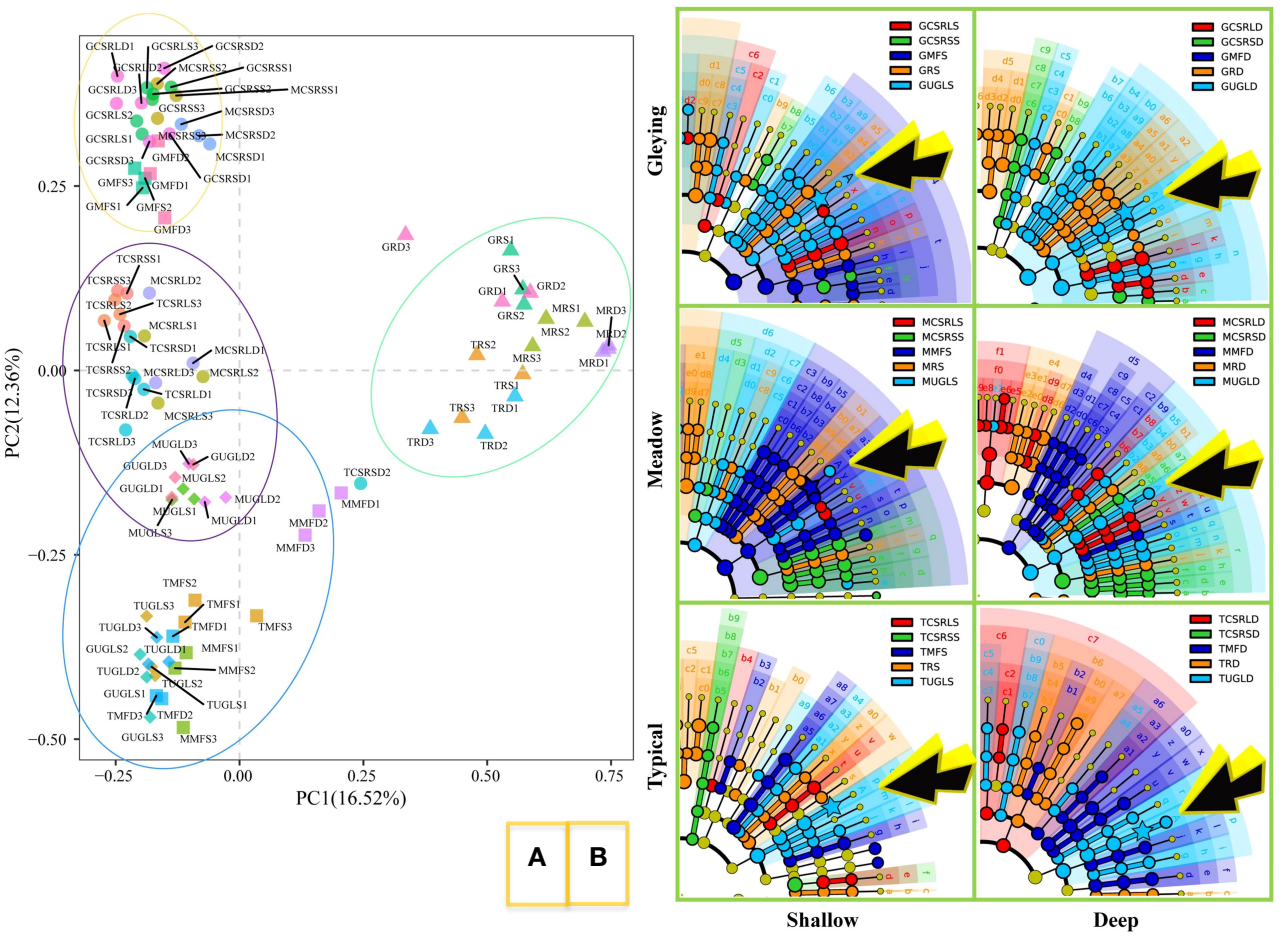
Effects of farming activities on soil bacterial communities. **(A)** Principal component analysis based on operational taxonomic unit (OTU) level. Abbreviations are shown in **Table 1** and the numbers at the end of the abbreviations represent three replicate experiments. The rice soil was clearly distinguished from the other soils and OTU similarity was defined as 97%. **(B)** Evolutionary branching diagram for linear discriminant analysis of effect size (LEfSe). Control analyses were performed according to each of the three soil types and two depths. Arrow-pointing A represents *Acidobacteria* genus *RB41* and background colour represents significant differences.

Bacterial beta diversity analysis is shown in **Figure 2A**. The clustering results of principal component analysis based on OTU level (green ellipses) show rice cultivation as a very distinct category. The blue ellipse shows that uncultivated land and manmade forest are closer in distance. Most interesting is the orange ellipse, which contains the meadow and gleying subtypes of albic black soils but not the typical subtype.

The results of LEfSe for the different soil subtypes are shown in **Figure 2B**. *Acidobacteria* genus *RB41* in the gleying albic black soil differed between UGL and rice. The same occurred in the shallow of the typical and meadow albic black soils. Furthermore, *Acidobacteria* genus *RB41* differed between UGL and MF. Differences between *Bradyrhizobium* and *Streptomyces* were not detected.

### 3.2 Farming activities affect SOC

**Figure 3:**
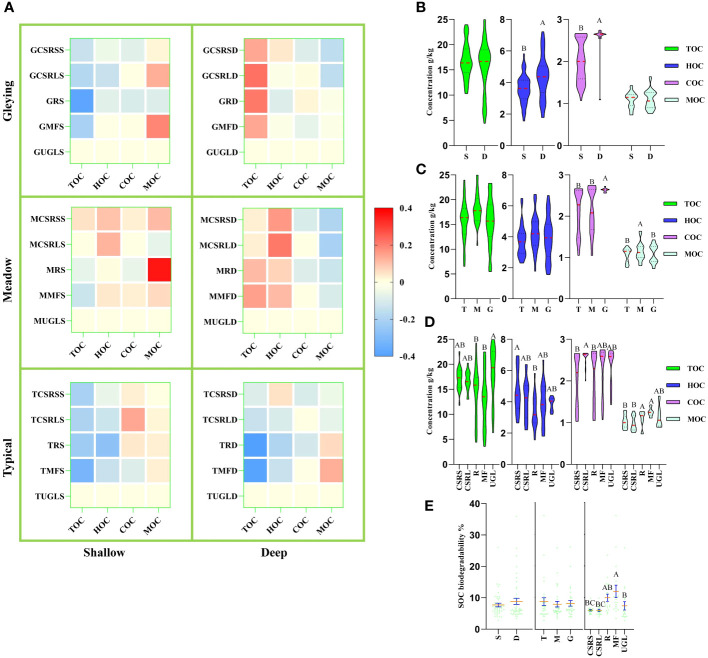
Effects of farming activities on soil organic carbon (SOC). **(A)** Heat map of relative changes in SOC: total, humus, carbohydrate and mineralizable organic carbon (TOC, HOC, COC and MOC, respectively) levels are plotted using uncultivated grassland/meadow (UGL) as a constant reference value; red colour represents an increase and blue represents a decrease. **(B–D)** Comparative violin plots of SOC concentration: **(B)** soil depth is divided into shallow (S) and deep (D); **(C)** soil type is divided into three subtypes – typical (T), meadow (M) and gleying (G); **(D)** land use is divided into five classes and the classification method is shown in **Table 1**. **(E)** Comparative analysis of SOC biodegradability: green dots represent sample values, orange lines represent mean values and blue bars represent standard errors; different letters in the graph indicate significant differences (*p<* 0.05).

**Figure 3A** shows that TOC concentrations were lower than UGL in the shallow group for both the MF and R treatments, and significantly lower than UGL overall for both treatments (**Figure 3D**). **Figure 3B** shows that HOC and COC concentrations were significantly lower in the shallow than the deep group. **Figure 3C** shows that the concentration of COC was significantly higher in the gleying subtype than in the typical and meadow subtypes and that the concentration of MOC was significantly higher in the meadow subtype than in the typical and gleying subtypes. **Figure 3E** similarly showed that the five land-use modes resulted in significant differences in SOC biodegradability.

### 3.3 Correlation between relative abundance of soil bacteria and SOC

**Figure 4:**
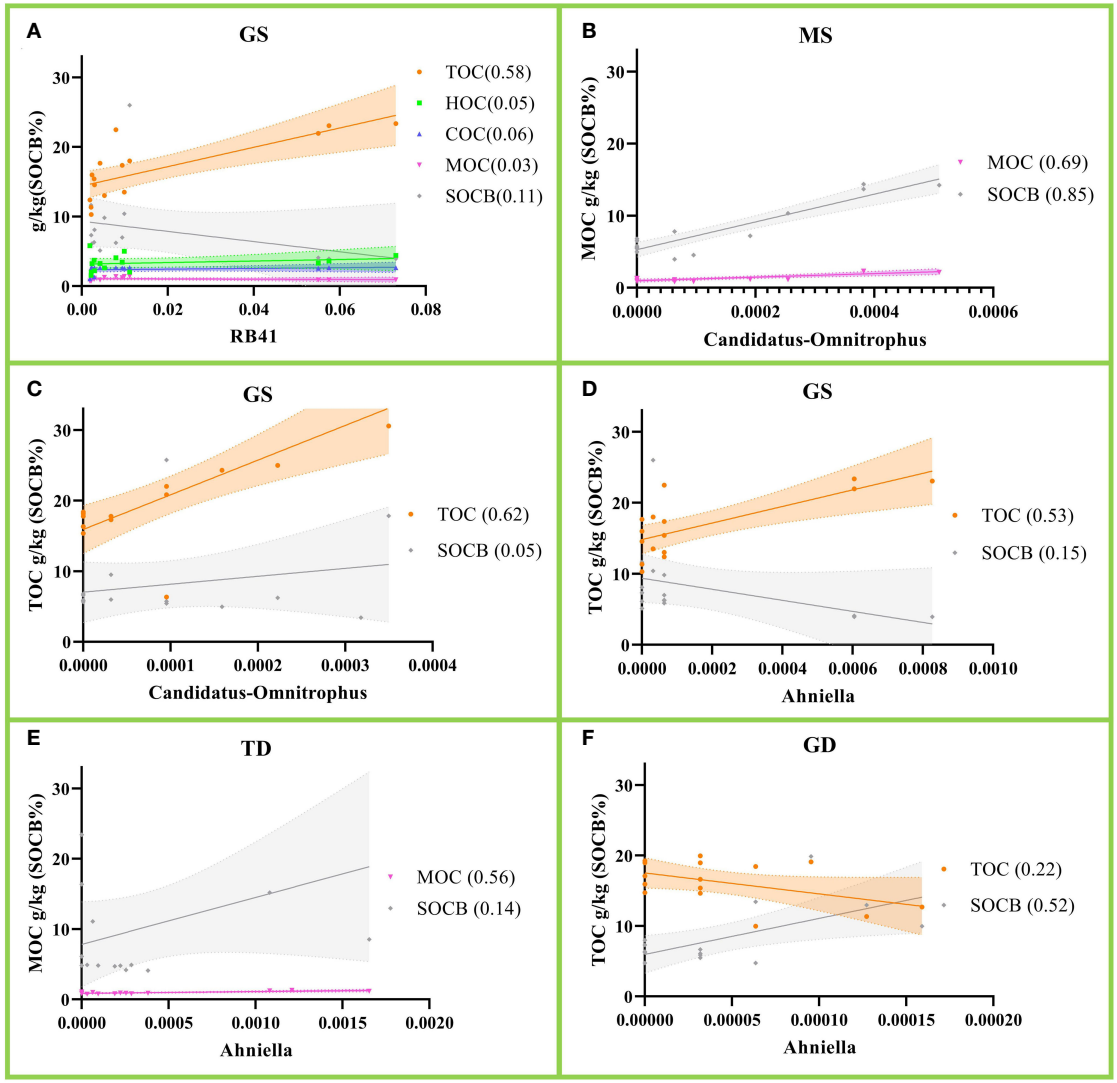
Correlation of relative abundance of soil bacteria with soil organic carbon (SOC). **(A)** Correlation of *RB41* with SOC in gleying shallow (GS) albic black soil; **(B, C)** correlation of *Candidatus-Omnitrophus* with SOC in meadow shallow (MS) and GS; **(D–F)** correlation of *Ahniella* with SOC in GS, typical deep (TD) and gleying deep (GD). The *r* ^2^ values are shown in parentheses and the dashed line represents the 95% confidence level.

**Figures 4A–D** show that *RB41*(*p*=0.001), *Candidatus-Omnitrophus*(*p*=0.0005) and *Ahniella*(*p*=0.002) are positively correlated with TOC in gleying shallow (GS) albic black soil. *Candidatus-Omnitrophus*(*p*=0.0001) and *Ahniella*(*p*=0.0013) are each positively correlated with MOC in meadow shallow (**Figures 4B** (MS) and **4E** (TD)). Notably, SOCB biodegradability in **Figures 4B, F** are each positively correlated with *Candidatus-Omnitrophus*(*p*<0.0001) and *Ahniella*(*p*=0.018). Our results clarify which genus of bacteria contributes most to soil carbon deposition.

## 4 Discussion

Most of the carbon sinks in the terrestrial natural environment are stored in the soil (Tao et al., 2023) and a large deposition of SOC reduces the concentration of CO_2_ in the atmosphere (Xiao et al., 2023). Due to human farming activities impacting carbon sequestration, soil carbon sinks have been altered (Wang et al., 2022). There is an equilibrium relationship in the carbon cycle in the natural state and human activities undoubtedly affect this balance (Ren et al., 2023). The purpose of this study is to investigate the effect of human activities on carbon deposition based on fine soil classification. At similar latitudes, different soil types with the same tillage practices can produce different changes in soil microorganisms (Liu et al., 2023d). However, none of the literature has examined soil microbial and SOC-related changes based on soil subtype.

### 4.1 Effects of human farming activities on soil microbial communities

Human farming activities effect on soil microbial communities. That are multifaceted include: fertilizer application (Guo et al., 2022; Ullah et al., 2023b), cultivation of different plants (Christel et al., 2024), pesticide use (Bhende et al., 2024) and land-use practices (Christel et al., 2024; Su et al., 2024). Our study found that rice cultivation led to an increase in anaerobic microorganisms, a phenomenon that was not explored in depth in this study because we are unsure of the contribution made by anaerobic microorganisms to soil carbon deposition.

The plough layer of the experimental land is 0-20cm deep, while plant roots can actually reach the 20-40cm depth. Additionally, arbuscular mycorrhizal fungi can extend the range reached by plant roots in the rhizosphere even further (Babalola et al., 2022). A study on microplastic influence on SOC showed that bacteria are affected differently by external factors at different depths of the soil layer (Liu et al., 2023c). The **Figure 2B** results of our study also indicate that the depth of the soil layer is an important condition that affects microorganisms. It is generally believed that the albic layer of albic black soils is not rich in nutrients and that the microorganisms living in it mainly rely on nutrients deposited from the top layer of black soil. However, the root system of plants can reach this depth and also provide the necessary nutrients for the microorganisms.

Different grassland-use patterns lead to changes in soil bacterial communities (Cao et al., 2023) Studies on different soil types have shown that soil microorganisms also differ among soil types under the same agricultural tillage conditions (Rodriguez-Echeverria et al., 2014; Raji and Thangavelu, 2021). A comparative study of different SOC levels between grasslands and agricultural cultivation revealed that long-term fertilization and irrigation have led to an increase in MBC (Li et al., 2019). We consider that the effect of farming activities on soil bacterial communities and SOC deposition is different for the three soil subtypes. The results of this study show that the orange ellipse analysed by principal component analysis contains the meadow and gleying subtypes of albic black soils but not the typical subtype. This suggests that it is essential to study the bacterial community variegation according to the fine soil classification. Our results **Figures 3C, E**, showing significant differences of COC and MOC concentrations in the typical, meadow and gleying subtypes, also support this consideration.

### 4.2 Farming activities affect SOC

SOC is a key indicator of soil quality. A high level of SOC means that more food can be produced (Boubehziz et al., 2024). On the one hand, soil absorbs carbon dioxide from the atmosphere and the organic fertilizers used also contain carbon sources. On the other hand, the output of food takes away part of the carbon source (Kong et al., 2024). This is a SOC balance process. At the UN Climate Change Conference of the Parties (COP21) in 2015, the task was proposed to increase soil carbon input, reduce soil carbon output, and increase soil organic matter by 0.4% every year (Gomez et al., 2024). SOC can be increased by agricultural management and improving soil quality (Ullah et al., 2023a). We have previously reported that the pattern of SOC changes in the rice cultivation process in meadow, black and planosol soils (Wang et al., 2022). The results involving meadow soil were similar to most of the results in this study; some of the different results may be due to the fact that the soil depths in this study were directly defined as 0–20 cm (shallow) and 20–40 cm (deep) and samples were collected without strictly following the tilth layer, plough pan layer and subsoil layer sampling (Wang et al., 2022). In addition, other research teams collecting 0–20 cm soil layer samples in river deltas reported that SOC concentrations have increased over the past 40 years (Liu et al., 2023a). However, the collection of 0–20 cm soil layer samples analysed showed that agricultural cultivation has led to a decrease in black soil SOC concentrations over the past 35 year (Wang et al., 2023). The **Figure 3D** results of this study showed that TOC concentrations for the MF and R treatments were significantly lower than for UGL, similar to the results reported by other teams. However, the TOC concentrations for UGL, CSRS and CSRL treatments were not significantly different. Compared to UGL, the four land use types are adopted by humans to obtain more agricultural products. MF and R reduce TOC. The comparison of the four land use types indicates that CSRS and CSRL did not reduce TOC. Therefore, they play a positive role in the deposition of soil carbon sink in terrestrial ecosystems.

### 4.3 Relationship between soil microorganisms and SOC deposition

According to the “soil microbial carbon pump” theory, it is believed that microorganisms are key factors in regulating the SOC pool (Kong et al., 2024). The cell walls of soil microorganisms are unstable and easily decomposable, playing a positive role in soil nutrient and material cycling (Fan et al., 2021). Lower SOC biodegradability indicates higher SOC stability. Furthermore, SOC biodegradability decreases with increasing latitude, suggesting that SOC becomes more stable with decreasing temperature (Liu et al., 2023b). The SOC biodegradability for the CSRS and CSRL treatments in our results **Figure 3D** suggests higher SOC stability.

Our results **Figure 4A** indicate a positive correlation between *Acidobacteria* genus *RB41* and SOC deposition. This is similar to the results reported by Stone (Stone et al., 2021). In addition, we found that *Candidatus-Omnitrophus* and *Ahniella* were positively correlated with TOC in GS albic black soil (**Figures 4C, D**). *Candidatus-Omnitrophus* occupies an important position in the underwater sediment (Williams et al., 2021; Suárez-Moo et al., 2022). There are few reports on *Ahniella* in soil and it has only been found in sorghum rhizobiome communities (Chou et al., 2023). The results of this study suggest that *Candidatus-Omnitrophus* and *Ahniella* are closely associated with SOC deposition. However, we are not sure whether SOC enhancement promotes *Candidatus-Omnitrophus* and *Ahniella* or whether *Candidatus-Omnitrophus* and *Ahniella* enhancement promotes SOC. In addition, it is not certain how SOC is transferred from *Acidobacteria* genus *RB41* to *Candidatus-Omnitrophus* and *Ahniella*.

### 4.4 limitations

Fungi and bacteria jointly influence soil carbon sequestration (Li et al., 2024). However, this study only investigated the role of bacteria in this process, without fully considering the promoting effects of fungi, which have a symbiotic relationship with bacteria and plants. Therefore, the study has certain limitations.

## 5 Conclusions

The effects of farming activities on soil bacterial communities and SOC deposition were different in three subtypes of albic black soil. Soil bacteria play a key role in the process of organic carbon deposition, with *RB41*, *Candidatus-Omnitrophus* and *Ahniella* being positively correlated with SOC deposition. Understanding the mechanisms and patterns of influence of soil bacteria on organic carbon deposition can help to improve soil fertility, protect the ecological environment and provide a scientific basis for soil carbon cycle management. Future research should focus on analysis of functional genes and the community structure of soil bacteria to reveal their key roles in the process of organic carbon deposition.

## Data availability statement

The original contributions presented in the study are included in the article/supplementary material, further inquiries can be directed to the corresponding author/s.

## Author contributions

QW: Writing – original draft, Writing – review & editing, Resources, Funding acquisition, Supervision. DZ: Writing – original draft, Resources. FJ: Writing – original draft, Writing – review & editing, Resources, Funding acquisition, Supervision. HZ: Writing – original draft, Resources. ZG: Writing – original draft, Writing – review & editing, Formal analysis, Supervision.
